# Understaffed and beleaguered: a national survey of chiefs of police about the post-George Floyd era

**DOI:** 10.1108/pijpsm-12-2023-0171

**Published:** 2024-08-14

**Authors:** Brandon del Pozo, Saba Rouhani, M.H. Clark, Danielle Atkins, Barbara Andraka-Christou, Kaitlin F. Martins

**Affiliations:** Rhode Island Hospital, Warren Alpert Medical School of Brown University, Providence, Rhode Island, USA; Department of Epidemiology, School of Global Public Health, New York University, New York, New York, USA; College of Community Innovation and Education, University of Central Florida, Orlando, Florida, USA; Askew School of Public Administration and Policy, College of Social Science and Public Policy, Florida State University, Tallahassee, Florida, USA; School of Global Health Management and Informatics, College of Community Innovation and Education, University of Central Florida, Orlando, Florida, USA; Division of General Internal Medicine, Rhode Island Hospital, Providence, Rhode Island, USA

**Keywords:** Retention, Policing, Morale, George Floyd, Law enforcement recruitment, Chiefs of police

## Abstract

**Purpose –:**

The 2020 murder of George Floyd resulted in challenges to policing in the United States of America, but little is known about how police chiefs perceive them. At the same time, chiefs of police wield great influence over public perceptions of crime and disorder, the state of their profession, the laws and policies that govern the conduct of police officers and municipal public safety budgets. It is therefore critical to understand how police perceive the changes to their profession post-Floyd.

**Design/methodology/approach –:**

This study surveyed a randomly selected national sample of 276 municipal chiefs of police. Items probed resignations, recruitment, efforts to defund departments, community support, officer morale, suspects’ likelihood of obeying lawful orders and career risks that could inhibit proactive police work. It examined associations between perceptions and Census Bureau region, length of tenure as chief, size of police department, population served and the urban or rural designation of the jurisdiction.

**Findings –:**

Chiefs overwhelmingly reported recruiting qualified candidates had become much harder, and the present risks of proactive police work encourage inaction. Chiefs of agencies in the Northeast perceived more challenges than those in the South. Respondents with more years of experience were less likely to perceive the current situation as dire. Approximately 13.5% reported an attempt to defund their department, 56.8% of which yielded some success. Our study suggests an increase in the number and scope of challenges perceived by chiefs of police. Results vary by region and police chief years of experience.

**Originality/value –:**

This study provides researchers and practitioners with the perspectives of chiefs about the post-Floyd era that influence their decisions, policies and initiatives.

## Introduction

The murder of George Floyd by Minneapolis police officers in the spring of 2020 catalyzed a historic period of national activism and protest, the effects of which are still being felt by police, municipalities, and communities over three years later. It generated calls to substantially reduce police department budgets and to reallocate their funds to alternative mechanisms for public safety (Eaglin, 2020; Jacobs et al., 2021; Su et al., 2021). It brought abolition—the elimination of police departments in their entirety—from a barely acknowledged fringe concept to one that captured the imagination of an increasing number of citizens and scholars (Kaba, 2020; Kaba and Ritchie, 2022; Purnell, 2021). These developments unfolded during the global COVID-19 pandemic, a time of considerable stress and uncertainty that heightened emotions, exacerbated a sense of crisis, and confounded Americans’ ability to parse the causes of significant declines in public safety across the nation (Ashby, 2020; Boman and Gallupe, 2020). While attempts to defund the police ultimately stalled in nearly all jurisdictions, including liberal cities initially believed to support the approach (Smith and Arango, 2021), and well-publicized efforts at police abolition were defeated at the polls by diverse constituencies (Bailey, 2021; Rao, 2020), the murder of George Floyd had a profound effect on the tenor of American policing, and the relationship between police officers and their communities (Lartey, 2022).

Research is emerging about the effects of these events on the police profession. Recent scholarship suggests considerably elevated levels of turnover in police agencies (Adams et al., 2023; Mourtgos et al., 2022) resulting in widespread staffing crises (PERF, 2023) compounded by pre-existing recruitment challenges (Wilson et al., 2010), significant increases in resistance by suspects during encounters with police in some cities (Boehme and Kaminski, 2023), and a significant short-term spike in firearms violence against police officers (Sierra-Arévalo et al., 2023). We do not, however, have a reliable understanding of how the nation’s police leadership has perceived and characterized these effects. While local and national news outlets have anecdotally reported on the sentiments of chiefs of police, there has been very little systematic research on how chiefs have perceived changes to the police profession since Floyd’s murder.

Information about how police chiefs perceive various challenges is critical for research and practice: it can provide focus for innovation and reform efforts, expose gaps in the present scholarship, and determine if the perceptions of police chiefs accord with both mainstream reporting and more systematic empirical observations. Police chiefs have a strong influence on local politics and budgets, and they can shape their constituencies perceptions about public safety and order. Their beliefs about the challenges of policing have the power to shape local laws, and policies, as well as set the tenor of police-community relationships. There is a diverse range of views about what policing should look like in the United States, from maintaining the status quo to the sweeping reforms demanded by ardent critics, and these efforts will be shaped in great part of the nation’s chiefs of police. It is therefore crucial to understand the factors that may motivate their decisions and statements. In an attempt to fill the existing gaps in our understanding of these matters, this study surveyed a randomly selected national sample of chiefs of police about changes in American policing since Floyd’s murder.

## Methods

In late 2021, we designed a survey to investigate municipal police chiefs’ views about substance use disorder and the US overdose crisis, in addition to their perceptions about changes in policing since the murder of George Floyd. Given the heterogeneity of their missions and political environments, we did not include executives who led state police, sheriff’s departments, or specialty departments such as university, park, or railroad police. The study was determined not to exceed minimal risk and was deemed exempt by the Lifespan Corporation’s IRB.

### Participants

Our sampling frame for random selection was drawn from the records of a commercial vendor (NPSIB, 2022) that maintains demographic data and contact information for the nation’s municipal police departments, including their current chief of police. From this universe of 11,757 municipal police departments, agencies reporting fewer than five sworn officers (*n* = 2,084) were excluded under the rationale that they would offer limited insights about staffing issues and the organizational complexities of policing. The remaining 9,673 departments were each assigned a random number and sorted in ascending order. The first 1,200 agencies were selected for our sample frame, which represented 12.3% of all municipal police departments in the United States with five or more officers. Initial paper surveys were mailed to each chief of police with a stamped return envelope with a unique identifier on it to monitor nonresponse. The survey was fielded in the winter and spring of 2022 in two waves, the second one limited to the nonrespondents of the first. Once data were collected and cleaned, they were stripped of all coded identifiers and effectively anonymized. Considering total material costs such as list procurement, printing, stationery, and postage, but excluding research effort, the cost per response in this study was approximately $18.57.

### Measurement

The survey included 37 items in total, 11 of which were specifically designed to capture perceived changes to the policing environment since the murder of George Floyd in May 2020. These 11 items measured perceptions of recruitment, retention, pre-retirement resignations, officer morale, efforts at defunding, and the behavior of citizens toward police.

Respondents were asked to rate their perception of the items using a sematic differential scale (i.e. a 1–7 visual analog scale with anchoring language), in addition to two yes/no questions about defunding. The 11 survey items used for this analysis and the demographic data we collected are presented in Table 1.

The item about proactive policing asked how chiefs thought their officers felt about its risks. There are many proactive policing techniques, and we did not know which ones each agency emphasized, so the item did not list specific practices. Rather, the item measured beliefs that could concern a range of police activities that would not have occurred but for police deciding to undertake them, in contrast with policing that emphasizes “reacting to particular events after they have occurred,” and “mobilizing resources based on requests coming from outside the police organization” (Majmundar and Weisburd, 2018, p. 30). For individual officers, these proactive measures typically include car and pedestrian stops, and investigating possible crimes in progress absent a specific community call for service. Similarly, instead of naming each aspect of the tumult that occurred during 2020, we asked chiefs what effect “the political and social events of 2020” had on their community’s safety. The goal was to understand the perceived state of policing rather than affix specific causes to it from among COVID, the murder of George Floyd, and the widespread unrest that followed.

Responses to each item were grouped according to how closely they aligned with their anchors regarding the series of propositions shown in Table 1, . We therefore grouped responses into five categories, depending on the valence of the proposition on the Likert scale: one for each of the two strongest perceptions (1 or 7), any non-neutral perception (1–3 or 5–7), and neutrality toward the proposition described (4). See Table 1.

We also generated an aggregate continuous score that we termed a beleaguerment scale by summing the first nine items of the survey. The index was developed to examine the perceptions of respondents about the comparative extent of the challenges they believed their police departments were facing in the post-George Floyd era. Items were reverse coded as necessary such that a final scale ranging from 7 to 63 points was created, where higher values suggested increased levels of beleaguerment. A Cronbach’s alpha of 0.70 suggested the items had a level of internal consistency appropriate for a scale undergoing initial development.

**Figure 1.**
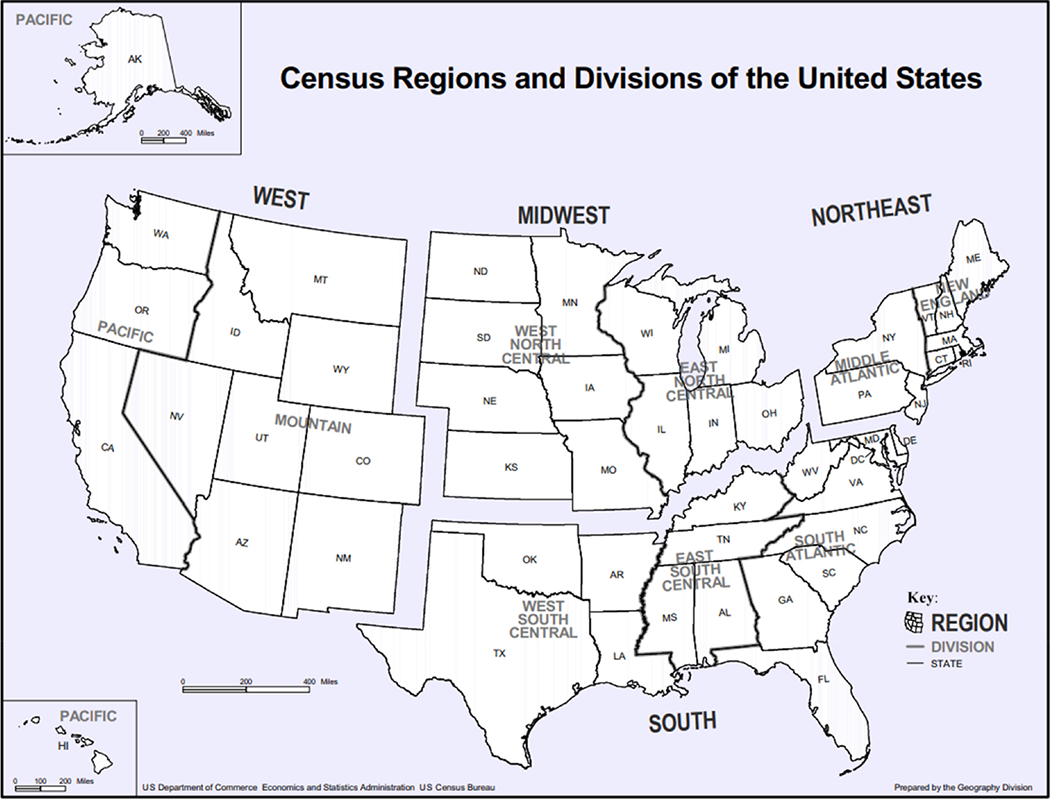
Census bureau regions of the United States **Note(s):** As a work of the US federal government, this image is in the public domain

Participants were then classified according to the US Census Bureau region of their agency (see Figure 1), agency size, urbanicity and population served. Using the zip codes of addresses provided for each agency’s headquarters in the sample, all entries were linked to a designation of urban (metropolitan or micropolitan) or rural (small town or rural).

### Data screening

In instances where a respondent did not follow instructions by circling only one answer on the Likert scale, we sought to include the data if the respondents’ intentions were sufficiently clear. If a respondent fully circled two consecutive numbers on a Likert scale, we entered their average. If they circled the space between two numbers but neither number itself, we entered the average of those two numbers. In a handful of cases where a respondent wrote “did not affect,” or “same,” we entered the middle value on the scale that coded a neutral response. We did not conduct a comparative analysis that eliminated these items because they reflected a respondent’s intention to provide us with data we were able to confidently impute. In cases where respondents did not enter their time in rank as the agency head, we used publicly available data to enter a value prior to fully anonymizing the responses. In a few cases we could not determine how long a person had worked in policing overall, and those cases were excluded from the relevant calculations. Omissions and indecipherable responses were considered missing and excluded from the analyses. All such steps were memorialized by notations in the dataset, and no visual patterns were observed among the entries concerned. To assess the possibility of nonresponse bias, analyses were conducted to compare several demographic covariates between those who did and did not respond to the survey. The findings of this nonresponse analysis are in the Results section below.

### Analyses

We examined associations between region, urbanicity, length of time served in the department (prior to becoming chief and as chief), and the ratio of the agency to population size with the score for each item in a fully adjusted ordinal logistic regression model with Likert scale response as the outcome (7 denoting the least desirable outcome). We then estimated unadjusted and adjusted associations between factors listed above and overall beleaguerment scale score (with 63 denoting the most beleaguerment across items) using a multiple linear regression model. All adjusted analyses accounted for Census region, years served prior to becoming chief, years served as chief, urbanicity, and the ratio of officers to the general population. To reduce the likelihood of type 1 errors, a Bonferroni correction was used for post hoc pairwise comparisons for region on individual survey items. To control the family-wise error rate, a Tukey’s HSD with a correction for multiple comparisons was used for all post hoc pairwise comparisons for region on beleaguerment scores. Analyses were conducted using Stata v.17 (StataCorp, 2021).

## Results

A total of 276 chiefs returned the survey, 194 in the first wave and 82 in the second, for a response rate of 23.0%. How this compares to other published research is taken up in the Discussion. Respondent characteristics, including demographic data, are presented in Table 2. The states with the highest number of respondents were Michigan (23), Pennsylvania (23), Texas (17), Ohio (15), Indiana (15), Illinois (15), and Wisconsin (14). The remaining states each had fewer than ten respondents. All states were sampled, and at least one chief from each state returned the survey except those from Vermont, South Dakota, Wyoming, Nevada, and Hawaii. The largest agencies to return the survey had nearly 500 sworn officers and served populations of up to 300,000 residents; the mean agency size was 32.3 (*SD* = 53.0), the mean population served was 16,733 (*SD* = 31,431), and the mean number of residents per officer was 464.9 (*SD* = 210.0). The respondents collectively represented police agencies serving 4.6 million US residents. Respondents served in agencies in the Northeast (21%), South (27.9%) Midwest (39.9%), and West (11.0%), and the majority (60.9%) of participants led agencies in metropolitan regions. The mean time on the force was 28.4 years in total, with 7 years as agency head (i.e. chief of police or equivalent title). We observed no significant differences between those who did and did not respond to the survey with respect to urbanicity, population size, or department size. However, there was a significant difference in response rate by region (*p* = 0.002): chiefs in the Midwest (29.4%) were more likely to return the survey than those in the South (18.9%). A great majority of respondents, 83.6% (*n* = 230), agreed recruiting well-qualified candidates to become police officers had become more difficult since the murder of George Floyd, with 45.1% (*n* = 124) of respondents strongly agreeing. Slightly more than a third of respondents reported an increase in pre-retirement resignations (35.1%, *n* = 95), while a majority reported no change (55.4%, *n* = 150). Of the responding chiefs, 80.1% (*n* = 221) felt events beyond their control affected people’s attitudes toward police, with 14.9% (*n* = 41) strongly agreeing. A majority of respondents agreed that “these days, officers feel proactive policing carries risks to their career that encourage them not to take action” (60.7%, *n* = 167), and over half (54.4%, *n* = 150) felt that since the murder of George Floyd, suspects were more likely to disobey lawful orders, although 30.4% (*n* = 84) reported people were now no more or less likely to do so. More than one-third of respondents, 40.9% (*n* = 112), reported that morale was lower since George Floyd was killed, 32.9% (*n* = 90) reported no change, and 26.3% (*n* = 72) reported that it was higher. Very few chiefs perceived that community support for police had strongly decreased since Floyd’smurder (9.8%, *n* = 27); rather, 27.3% (*n* = 75) indicated community support had remained the same, and 62.9% (*n* = 173) perceived it had strongly increased. Nearly half of respondents felt the political and social events of 2020 made people in their communities less safe (47.8%, *n* = 132), while most of the remainder felt there had been no change (37.0%, *n* = 102). Only 13.5% (*n* = 37) of respondents reported an attempt to defund their police department, but more than half of those respondents (56.8%, *n* = 21) reported the attempt had been met with at least some success. Descriptive statistics for all survey responses are presented in Table 3.

There were significant differences in the responses by region, urbanicity, and years served as a chief (Table 4). For Census Bureau region, we set the Northeast as the reference, as it elicited the strongest responses about the challenges they faced since the murder of George Floyd. On a scale of 1–7, respondents from the Southern Census Bureau region had significantly lower odds of reporting challenges than those serving in the Northeast on several items: morale (Odds Ratio (OR) 0.42, *p* < 0.01), recruitment (OR 0.48, *p* < 0.01), and the likelihood a person would disobey a lawful order (OR 0.57, *p* < 0.10). Likewise, chiefs responding from the Western Census Bureau region reported significantly fewer problems with recruiting officers than those in the Northeast (OR 0.39, *p* < 0.01), and chiefs responding from the Midwest region felt their constituents had a better grasp of the complexities of policing (OR 0.57, *p* < 0.10) than did those in the Northeast. No other significant differences between regions were identified in pairwise comparisons (data not shown).

Compared to metropolitan/micropolitan areas, chiefs in small towns and rural areas were significantly more likely to find it harder to recruit qualified candidates since the murder of George Floyd (0.49, *p* < 0.05). When pairwise comparisons were explored, there were no significant differences between any regions on any item except for recruitment. After a Bonferroni correction for multiple comparisons, post hoc pairwise comparisons showed that relative to respondents in the Southern Census Bureau Region, those in the Midwest, where George Floyd was murdered, had increased odds of reporting recruitment challenges (OR 1.97, *p* < 0.1).

How long a responding chief had served in total as a police officer did not significantly affect their perceptions of the policing challenges examined by our instrument, and neither did the size of the agency they led. However, length of time serving as a chief of police was significantly associated with lower odds of reporting several types of challenges post-Floyd. Specifically, respondents with longer tenures as chief reported higher morale (−0.03, *p* < 0.05), fewer resignations (−0.02, *p* < 0.10), a lower likelihood that suspects would disobey lawful orders (−0.02, *p* < 0.10), and more confidence in the public’s ability to grasp the complexity of policing (−0.04, *p* < 0.01).

### Beleaguerment

The mean beleaguerment score for all respondents was 40.8 (95% CI 40.0, 41.6) on a scale ranging from 7 (no comparative beleaguerment) to 63 (very high beleaguerment). The mean beleaguerment score for the Northeast Census Bureau region was 41.8 (95% CI 40.3, 43.4), for the South was 39.7 (95% CI 38.2, 41.1), for the Midwest was 41.2 (95% CI 39.9, 42.4), and for the West was 40.4 (95% CI 37.8, 43.0). After accounting for other predictors, Southern chiefs had significantly lower beleaguerment scores than their counterparts in the Northeast. No other significant differences between regions were identified in pairwise comparisons. The number of years a respondent had spent as a chief of police had a significant inverse relationship with mean beleaguerment score across the sample (unadjusted coefficients: 0.16, *p* < 0.01; adjusted: 0.17, *p* < 0.01), suggesting that chiefs with more experience perceived fewer negative changes in policing in their jurisdictions. Adjusted and unadjusted estimates for the associations between all characteristics and beleaguerment are shown in Table 5.

## Discussion

To our knowledge, this is the first study to survey a randomly selected national sample of US chiefs of police about changes to the profession in the aftermath of George Floyd’s murder. Our results corroborate several recent narratives about increased challenges in policing, including reports of challenges in recruitment that are strongly felt and widespread, and that suspects have become more likely to resist the police. Nearly half of the chiefs who responded to our survey perceived the events of 2020 as having left their communities less safe, with just over 60% reporting that their officers perceive proactive policing has become risky enough to deter taking action. Our findings with respect to officer willingness to engage in proactive police work corroborate research by Gau et al. (2022), possibly reflecting elevated concerns among police officers about the career-damaging outcomes of negative public and media attention. Such findings can be interpreted differently, since mainstream media accounts suggest community perceptions of proactive policing are heterogeneous. Some members of the public view proactive policing as inherently inequitable or problematic, while others feel it is necessary to prevent crime and preserve public order, and therefore desirable. If chiefs of police are correct about declines in proactive policing owing to officer perceptions about its risks, it suggests communities should measures the extent of such reductions, and assess the results of these changes in practice. Whether they are a net positive or negative is one of the most essentially contested aspects of contemporary public safety.

As municipalities emphasize the need for police officers who can use excellent judgment under challenging circumstances, the recruitment difficulties cited by chiefs should be of particular concern (del Pozo and Moskos, 2023). To boost staffing amidst these labor shortages, agencies are considering lowering their hiring standards (Klemko, 2023b), possibly exposing communities to greater risks of officer misconduct and abuse (Johnson, 2023; Lucas, 2023). Some have argued that the killing of Tyre Nichols by Memphis, Tennessee police officers could be attributed in part to such relaxed hiring standards (Klemko, 2023a). This signals the urgent need for research into these approaches and their consequences. Given the critical need to recruit qualified officers and the consequences of lowering standards, agencies might consider specifically appealing to women. They are policing’s principal untapped demographic, making up only about 13% of the policing’s sworn workforce. Reducing this disparity may be a way to bring a new source of highly qualified applicants into the nation’s police departments. The 30x30 Initiative supports agencies in this effort, providing research about how to effectively recruit, hire and retain women in policing, and providing a repository of evidence that women can improve the performance of police departments in several dimensions (McGough and Roman, 2024). Beyond this, agencies might evaluate the innovations in recruitment recently cataloged by the Police Executive Research Forum (2023) and utilize the ones most suitable for their own contexts.

Our results found regional differences in perceived challenges, with chiefs in the Northeast reporting facing the most acute challenges and those in the South facing the least. The cause of these regional differences is unclear, suggesting the need to research regional variance in police-community relations and local politics. We also found that most chiefs felt their departments were affected by external events beyond their control, possibly due to experiencing negative backlash from George Floyd’s murder despite being located in a different jurisdiction. Research might examine the extent to which communities across the nation feel that while Floyd was murdered in Minneapolis, the conditions for a Floyd-type incident exist in their own community.

Despite widely portrayed vitriol toward police in the form of slogans such as “All Cops Are Bastards” (Groundwater, 2020; Poulter, 2020), we found that 90% of chiefs felt that community support for their police either stayed the same or increased since the murder of George Floyd. It is possible that police chiefs in our sample consider such critics as more extreme or otherwise different from the typical constituent in their jurisdiction, or perceived that the occupational effects of Floyd’s murder were acutely felt in their departments, but their own communities were not the source of them. The fact that a great majority of our respondents did not report changes in community support suggests recruitment challenges are caused by other factors. Significantly more research, especially including qualitative work, is needed to understand the causes of recruitment challenges in policing.

Although there was media attention given to efforts at defunding and abolishing police departments, only 13% of respondents in our survey indicated their agency had experienced defunding efforts; approximately half of those respondents indicated at least some changes had occurred as a result. Our findings about the relatively limited extent of defunding efforts confirm those reported by other sources (Freiss, 2022). Significantly more research is needed to understand policing and community factors associated with defunding efforts, including those associated with successful efforts.

The findings here may provide the reasons behind anecdotal reporting that many chiefs are electing to end their tenure early, and some municipalities struggling to find high-caliber replacements (Greenblatt, 2020; Yancey-Bragg, 2020). Notably, respondents with longer tenure serving as police chiefs had lower overall levels of beleaguerment, including being less likely to report problems with morale, resignations, and the belief suspects are now more likely to disobey lawful orders. These findings about the inverse relationship between years as chief and level of beleaguerment have at least two potential explanations, both worthy of further research. First, longer-serving chiefs may have experienced more cycles of protest and reform in policing, and as a result are better able to contextualize the Floyd protests while leading their agencies with a calmer and steadier hand, leading to fewer challenges for the police agency. Another possibility is that the number of years serving as chief may be positively associated with community satisfaction with the local status quo in policing, which manifested as a more muted reaction to Floyd’s murder and its fallout, in turn resulting in fewer perceived challenges for such chiefs.

### Limitations

This study has limitations. While drawn randomly, the initial sample was not geographically stratified or were the results weighted, so while it was nationally representative of police departments overall, our sample was not representative by state or region considering the heterogeneity in the number of municipal police departments in the USA. The regional differences observed in our analyses should be interpreted with caution given the greater proportion of nonresponse from agencies in the Southern region of the United States than others. Likewise, although we recommend research at the regional level to better understand what we have observed here, our dataset does not have the subsample sizes necessary to undertake it. It can only suggest the direction of such research and provide exploratory hypotheses.

Our overall response rate of 23% is within the range seen in peer reviewed police survey work (Nix et al., 2019). In comparison, the Police Executive Research Forum recently surveyed its membership of 1,068 chiefs and other law enforcement executives about the staffing challenges in American policing using multiple waves of emails (PERF, 2023). The effort yielded 266 completed questionnaires, amounting to a response rate of 24.9% drawn from a convenience sample of police executives affiliated with this membership organization. In other published research, a link to a survey about executives’ receptivity to research was emailed to Oregon’s chiefs of police and sheriffs, yielding a 26.0% (*n* = 45) response rate (Telep and Winegar, 2015). This result was comparable to a contemporary study that sent surveys by mail and online to the chiefs in all 340 agencies reporting a sudden cardiac death in the line of duty from 1984–2010, with a 27% (*n* = 93) response rate (Korre et al., 2014). Finally, an online survey about crisis intervention training that targeted 746 chiefs of police and sheriffs in Georgia yielded a 27% (*n* = 204) response rate (Compton et al., 2015). Not only did our study have response rates comparable to those of previously published research surveying chiefs of police, but our sample may better represent chiefs in the US considering its sample size and random sampling approach.

It is also important to note this study captures assertions made by chiefs of police that may not be supported by empirical data. Some items made direct empirical inquiries, such as whether efforts were made to defund their departments, and if they were successful. The others, however, asked respondents to report their perceptions about contested beliefs. For example, our item about proactive policing both concerns a general concept and furthermore asks chiefs to opine on what their police officers feel about it. We have no way of knowing if these perceptions are accurate, or if they are what a respondent would like to portray as the prevailing situation, only that they were reported by a survey directed toward chiefs of police and in most cases conform to both anecdotal reporting in the media and emerging empirical research. Insofar as subjective perceptions influence the decisions and actions of a person charged with the leadership of a police department, they remain highly relevant to understanding the current state of American policing. At root, the responses can be viewed as what chiefs of police want researchers, and presumably the public, to think they perceive. That said, data were collected during the early winter of 2022, and both the perceptions of chiefs of police and the underlying facts have continued to change and evolve. Ultimately, policing in the United States is an intensely local practice, suggesting the need for researchers to understand the perceptions of chiefs of police at the municipal and metropolitan levels, assess if they align with empirical observations, and construct evidence-based policy responses accordingly.

## Conclusion

In the spring of 2020, the murder of George Floyd spawned calls for the defunding and abolition of police institutions and sent the police profession into a state of disarray. Since then, with a few notable exceptions (Siegel and Rappleye, 2021; Venkataramanan, 2020) abolition has not happened, and defunding was never a serious concern in most agencies (Freiss, 2022), assertions supported by the results of our study. At the same time, the people who lead the nation’s police departments report perceptions that should concern both constituents satisfied with policing in the USA and those seeking considerable reforms. Of note, our results highlight the ongoing difficulty of hiring and retaining a qualified workforce and suggest that the perceived risks of proactive policing are widespread. This national survey points toward a need to understand the challenges expressed by the nation’s municipal police leaders, whether these perceptions are consistent with empirical evidence, and what their consequences will be for policing and public safety. It confirms calls for research about how to effectively address these concerns while preventing future acts of police brutality and the glaring racial disparities they often reflect.

## Figures and Tables

**Table 1.** Survey items

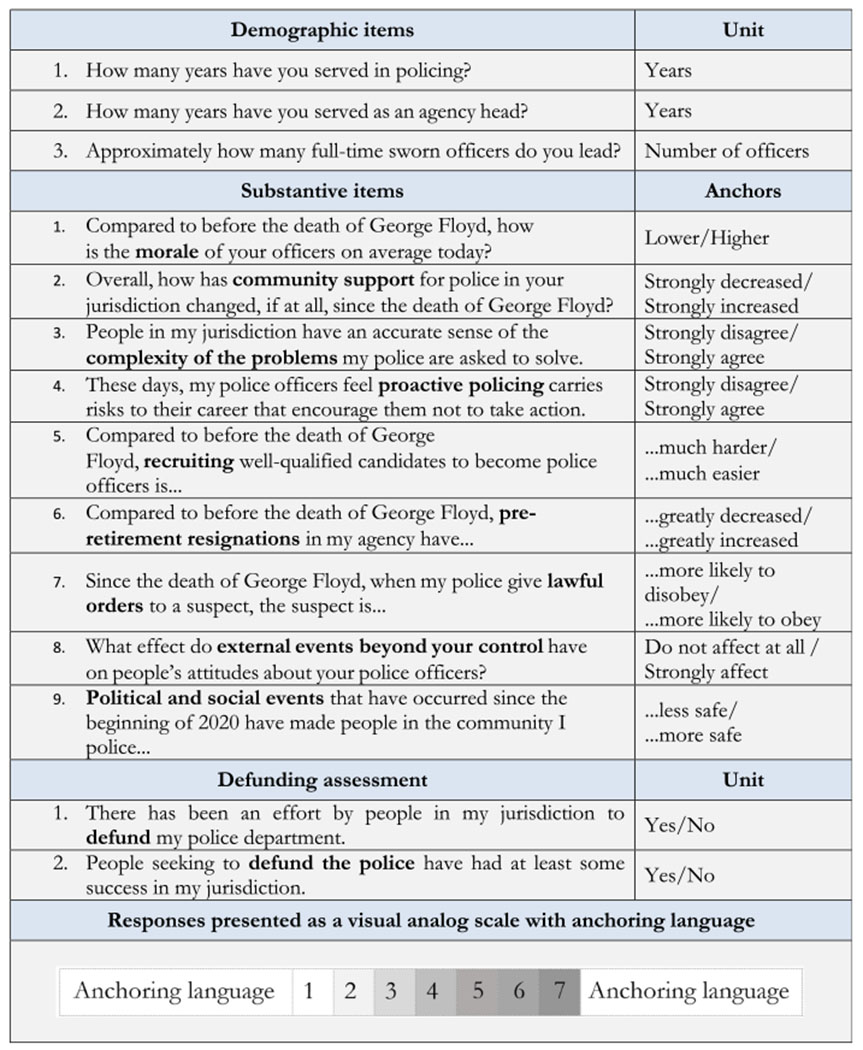

**Source(s):** Table and all contents by the authors

**Table 2.** Respondent characteristics, *N* = 276

*By Census Bureau region*	*N*(%)
Northeast	58 (21.0%)
South	77 (27.9%)
Midwest	110 (39.9%)
West	31 (11.2%)
*Urbanicity*	
Metropolitan	168 (60.9%)
Micropolitan	46 (16.7%)
Small Town	43 (15.6%)
Rural	19 (6.9%)
*Urbanicity – binary*	
Metropolitan/Micropolitan	214 (77.5%)
Small town/Rural	62 (22.5%)
*Population served*	*Mean (SD)*
Mean Population	16,733.1 (31431.3)
*Staffing*	
Sworn headcount	32.3 (53.0)
Residents per officer	464.9 (210.0)
*Respondent tenure*	
Total years served	28.41 (8.6)
Years as agency head	7.02 (6.67)
Total years served at lower ranks	21.5 (7.7)

**Source(s):** Table and all contents by the authors

**Table 3.** Response analysis by item

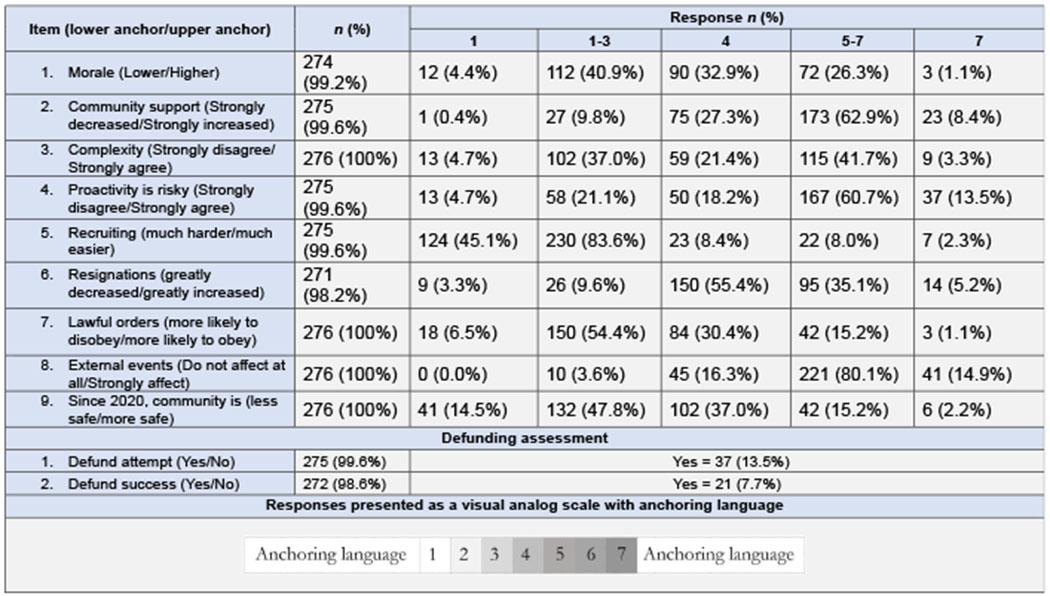

**Note(s):** *N* = 276

**Source(s):** Table and all contents by the authors

**Table 4.** Associations between item responses, demographics, and agency characteristics

Census regions	Morale^+^	Support^+^	Complexity^+^	Proactivity	Odds ratios Recruiting^+^	Resignations	Disobey^+^	External	Less Safe^+^
Northeast					Reference				
South	0.42***	1.15	1.00	0.70	0.48***	0.57	0.57*	0.79	0.68
Midwest	0.78	0.94	0.57*	1.04	0.94	1.12	0.83	1.01	0.79
West	0.59	1.08	0.92	0.53	0.39***	1.24	0.93	1.60	0.71
*Urbanicity*									
Metro/Micropolitan					Reference				
Small Town/Rural	0.96	0.97	1.02	0.92	1.59*	1.17	0.73	0.99	0.80
*Tenure*									
Years as chief	0.97*	0.98	0.95***	0.99	0.96**	0.97*	0.98	0.98	1.02
Years not as chief	0.99	1.00	0.99	1.02	0.98	1.01	0.99	1.05	1.02
*Staffing*									
Residents per officer	1.02	0.97	0.97	0.94	1.02	0.96	0.96	0.98	1.04

**Note(s):**

+ Identifies items reverse coded such that a higher score denotes a less desirable outcome or state of affairs among chiefs

* denotes significance at *p* < 0.10

** denotes significance at *p* < 0.05

*** denotes significance at *p* < 0.01

**Source(s):** Table and all contents by the authors

**Table 5.** Beleaguerment scale

Census region	Unadjusted coefficient	Adjusted coefficient
Northeast: 41.8 (95% CI 40.3, 43.4)	Reference	Reference
South: 39.7 (95% CI 38.2, 41.1)	−2.18	−2.58**
Midwest: 41.2 (95% CI 39.9, 42.4)	−0.68	−0.93
West: 40.4 (95% CI 37.8, 43.0)	−1.44	−1.62

Metropolitan/Micropolitan	Reference	Reference
Small Town/Rural	−0.1	0.04
*Chiefs’ tenure*		
Years as chief	−0.16***	−0.17***
Years at lower ranks	0.04	−0.002
*Staffing*		
Ratio of population to officers	−0.01	−0.14

**Note(s):** *N* = 276.

* denotes significance at *p* < 0.10

** denotes significance at *p* < 0.05

*** denotes significance at *p* < 0.01

**Source(s):** Table and all contents by the authors
